# Impact of COVID-19 and effects of BNT162b2 on patient-reported outcomes: quality of life, symptoms, and work productivity among US adult outpatients

**DOI:** 10.1186/s41687-022-00528-w

**Published:** 2022-12-05

**Authors:** Manuela Di Fusco, Xiaowu Sun, Mary M. Moran, Henriette Coetzer, Joann M. Zamparo, Laura Puzniak, Mary B. Alvarez, Ying P. Tabak, Joseph C. Cappelleri

**Affiliations:** 1 Health Economics and Outcomes Research, Pfizer Inc., New York, NY USA; 2 CVS Health, Woonsocket, RI USA; 3 MDSCA Vaccines, Pfizer Inc., Collegeville, PA USA; 4 Field Medical Outcomes and Analytics, Pfizer Inc., New York, NY USA; 5 Statistical Research and Data Science Center, Pfizer Inc., Groton, CT USA

**Keywords:** COVID-19, SARS-CoV-2, HRQoL, WPAI, Quality of life, COVID-19 symptoms, BNT162b2, Humanistic

## Abstract

**Background:**

Although there is extensive literature on the clinical benefits of COVID-19 vaccination, data on humanistic effects are limited. This study evaluated the impact of SARS-CoV-2 infection on symptoms, Health-Related Quality of Life (HRQoL) and Work Productivity and Impairment (WPAI) prior to and one month following infection between individuals vaccinated with BNT162b2 and those unvaccinated.

**Methods:**

Subjects with ≥ 1 self-reported symptom and positive RT-PCR for SARS-CoV-2 at CVS Health US test sites were recruited between 01/31/2022 and 04/30/2022. Socio-demographics, clinical characteristics and vaccination status were evaluated. Self-reported symptoms, HRQoL, and WPAI outcomes were assessed using questionnaires and validated instruments (EQ-5D-5L, WPAI-GH) across acute COVID time points from pre-COVID to Week 4, and between vaccination groups. Mixed models for repeated measures were conducted for multivariable analyses, adjusting for several covariates. Effect size (ES) of Cohen’s d was calculated to quantify the magnitude of outcome changes within and between vaccination groups.

**Results:**

The study population included 430 subjects: 197 unvaccinated and 233 vaccinated with BNT162b2. Mean (SD) age was 42.4 years (14.3), 76.0% were female, 38.8% reported prior infection and 24.2% at least one comorbidity. Statistically significant differences in outcomes were observed compared with baseline and between groups. The EQ-Visual analogue scale scores and Utility Index dropped in both cohorts at Day 3 and increased by Week 4 but did not return to pre-COVID levels. The mean changes were statistically lower in the BNT162b2 cohort at Day 3 and Week 4. The BNT162b2 cohort reported lower prevalence and fewer symptoms at index date and Week 4. At Week 1, COVID-19 had a large impact on all WPAI-GH domains: the work productivity time loss among unvaccinated and vaccinated was 65.0% and 53.8%, and the mean activity impairment was 50.2% and 43.9%, respectively. Except for absenteeism at Week 4, the BNT162b2 cohort was associated with statistically significant less worsening in all WPAI-GH scores at both Week 1 and 4.

**Conclusions:**

COVID-19 negatively impacted HRQoL and work productivity among mildly symptomatic outpatients. Compared with unvaccinated, those vaccinated with BNT162b2 were less impacted by COVID-19 infection and recovered faster.

**Supplementary Information:**

The online version contains supplementary material available at 10.1186/s41687-022-00528-w.

## Background

The impact of the COVID-19 pandemic on the sustainability of quality of life of patients has been reported globally [1–4]. The prolonged multisystem symptoms associated with SARS-CoV-2 infection can negatively affect daily activities, ability to work, and social interactions, leading to poor health-related quality of life (HRQoL) [1–4].

Most of the studies assessing humanistic outcomes of COVID-19 infection have been limited to inpatients [1, 2, 5], were conducted outside of the US, or focused on specific disease states and organ-specific functions [6–8]. There are limited studies measuring the health-related well-being of non-hospitalized COVID-19 patients [7–9].

The introduction of COVID-19 vaccination has significantly impacted the COVID-19 response, and evidence regarding the efficacy, safety and effectiveness of vaccination is extensive [9]. However, there is limited research on the potential benefits of vaccination on physical, mental, social, emotional functioning and economic well-being. It has been increasingly recognized that such aspects of health and well-being are best described by the patients themselves through Patient-Reported Outcomes (PRO), without amendment or interpretation of the patient's response by a clinician or anyone else [10–12]. Both generic and disease-specific PROs have been widely used in vaccine research to measure the patient experience of disease-related symptoms, disease impact and HRQoL [12]. Leveraging a US national retail pharmacy SARS-CoV-2 test database and using validated PRO measures (PROMs), the objective of this study was to address such evidence gaps by assessing COVID-19 symptoms, HRQoL and WPAI prior to through one month following SARS-CoV-2 infection in outpatients, and compared results between unvaccinated individuals and those vaccinated with BNT162b2.

## Methods

### Study design and participants

The source population consisted of individuals testing for SARS-CoV-2 at one of ~ 5000 CVS Health test sites across the US. As part of the registration process for scheduling a SARS-CoV-2 test at CVS Health, individuals are required to complete a screening questionnaire including demographics, symptoms, comorbidities, and vaccination status. The screening variables and RT-PCR test results are loaded in an analytic dataset, where ~ 80–90% of test results are reported within 2–3 days. Leveraging this analytic platform, this study was designed as a prospective survey-based patient-reported outcomes study targeting adults ≥ 18 with a positive RT-PCR test result and self-reporting at least one symptom. Asymptomatic individuals were excluded. These criteria were chosen to balance a broad characterization of SARS-CoV-2 symptoms with representativeness of the working-age population for WPAI analyses.

**Fig. 1:**
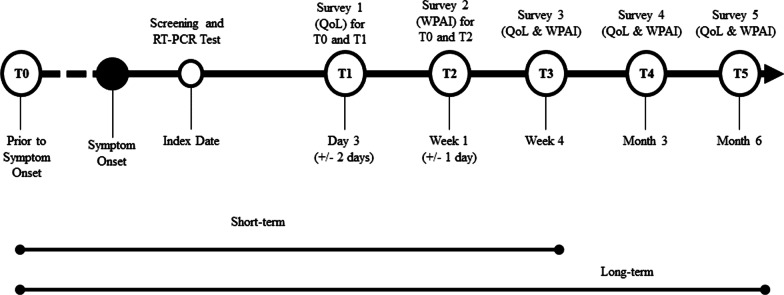
Study design. *Notes* QoL refers to the EQ-5D-5L survey

These individuals were emailed an invitation as soon as the test results became available, no later than 4 days from testing. The email invitation directed the potential participants to an e-consent website to learn about the study, survey schedule and informed consent. Figure 1 summarizes the study design. Recruitment of participants was carried out between 01/31/2022 and 04/30/2022 (Ct.gov NCT05160636).

### Data sources and variables

#### Baseline characteristics and symptoms

Baseline characteristics of the participants were obtained via the CVS Health pre-test screening questionnaire. These included self-reported demographics, comorbidities (including immunocompromised status), COVID-19 vaccination history, social determinants of health including the Social Vulnerability Index (SVI), work and/or residency in a high-risk or healthcare setting, and symptoms. The list of COVID-19 symptoms was based on the set of symptoms defined by the Centers for Disease Control and Prevention (CDC) [13].

#### Exposure groups

Immunocompetent participants were considered fully vaccinated with BNT162b2 if they self-reported receipt of 2 doses ≥ 14 days of SARS-CoV-2 testing. They were considered partially vaccinated if reporting receipt of a single dose and boosted if reporting receipt of 3 doses. Participants self-reporting an immunocompromising condition and receipt of 3 doses were considered fully vaccinated and boosted if reporting 4 doses. Participants were considered unvaccinated if they did not report any COVID-19 vaccine dose prior to testing. Heterologous schedules were excluded.

#### HRQoL

To assess HRQoL, we used the EQ-5D-5L questionnaire [14, 15]. On the day of enrollment, consented participants completed the EQ-5D-5L questionnaire twice, using two versions: a modified version where all the questions were past tense to retrospectively assess pre-COVID-19 baseline QoL, and the standard version in present tense to assess current QoL. To minimize responder bias, the order of administration of the two versions was random. Subsequent completion was requested at one month (Fig. 1). The EQ-5D-5L results at each time point were converted into the Utility Index (UI) using the US-based weights by Pickard et al. [15, 16].

#### Work productivity and activity impairment

To measure impairments in both paid work and unpaid work, we used the Work Productivity and Activity Impairment General Health V2.0 (WPAI:GH) measure [17, 18]. Participants were asked to complete this questionnaire twice, seven days after their RT-PCR test: once referencing seven days prior to COVID-19 symptom onset and an additional assessment referencing the past seven days. Similar to the EQ-5D-5L, subsequent completion of the WPAI was requested at one month (Fig. 1). Four WPAI scores were computed at each time point: percent of worktime missed (absenteeism), percent of impairment while working (presenteeism), percent of work productivity loss (considering both absenteeism and presenteeism), and percent of activity impairment. Only employed subjects were included for work productivity analyses.

#### Post-COVID 19 symptoms and vaccination status update

To supplement the pre-test screening questionnaire and enable the collection of on-going or new symptoms after the acute phase, participants were sent an additional survey four weeks following the test asking to complete a checklist of COVID-19 related symptoms based on the CDC list [19], To confirm vaccination status, participants’ subsequent responses to vaccination date questions were compared with their index responses; if responses did not match, the information was queried and adjudicated, and the latest information was typically used.

### Statistical analysis

Descriptive statistics were used to analyze participant characteristics at baseline. Continuous variables were described using means and standard deviations. Categorical variables were reported using number and percentage distributions. For continuous variables, t-tests were used to test difference in means. For categorical variables, chi-square tests and Fisher’s exact tests were used to test differences between groups [20]. P values were all two-sided and not adjusted for multiplicity. Mixed models for repeated measures (MMRM) [21] were used to estimate the magnitude of COVID-19 impact on HRQoL and WPAI over time. Assessment time was fitted as a categorical covariate and a repeated effect (repeated by subject). Least squares mean (LS mean) and standard errors of PRO scores for each time point of assessment were calculated. Per guidelines, no adjustment was made for missing data when scoring the EQ-5D-5L UI and WPAI [14, 18]. Missing data at each timepoint were not imputed. All available data were included in the analysis.

Cohen’s d, or a variation of it, was calculated to assess the magnitude of score change from baseline within the BNT162b2 vaccinated cohort and, separately, the unvaccinated cohort, as well as the difference between BNT162b2 and unvaccinated cohorts [22, 23]. Specifically, within-cohort effect size (ES) was calculated as mean change from baseline to follow-up, divided by the standard deviation of change scores from baseline to follow-up. Between-cohort ES was calculated as the difference in mean changes from baseline between cohorts, divided by the pooled standard deviation of change scores. Values of 0.2, 0.5, and 0.8 standard deviation (SD) units represent small, medium, and large ES, respectively. These cut-off estimates have been widely used to establish important differences in HRQoL studies [24]. As such, we considered the magnitude of (standardized) effect sizes of at least 0.20 SD units as important or meaningful differences in gauging the magnitude of within-patient change and between-group differences. All data obtained were collected and analyzed with SAS Version 9.4 (SAS Institute, Cary, NC). The study followed the Strengthening the Reporting of Observational Studies in Epidemiology (STROBE) reporting guideline [25].

## Results

### Baseline characteristics

**Fig. 2:**
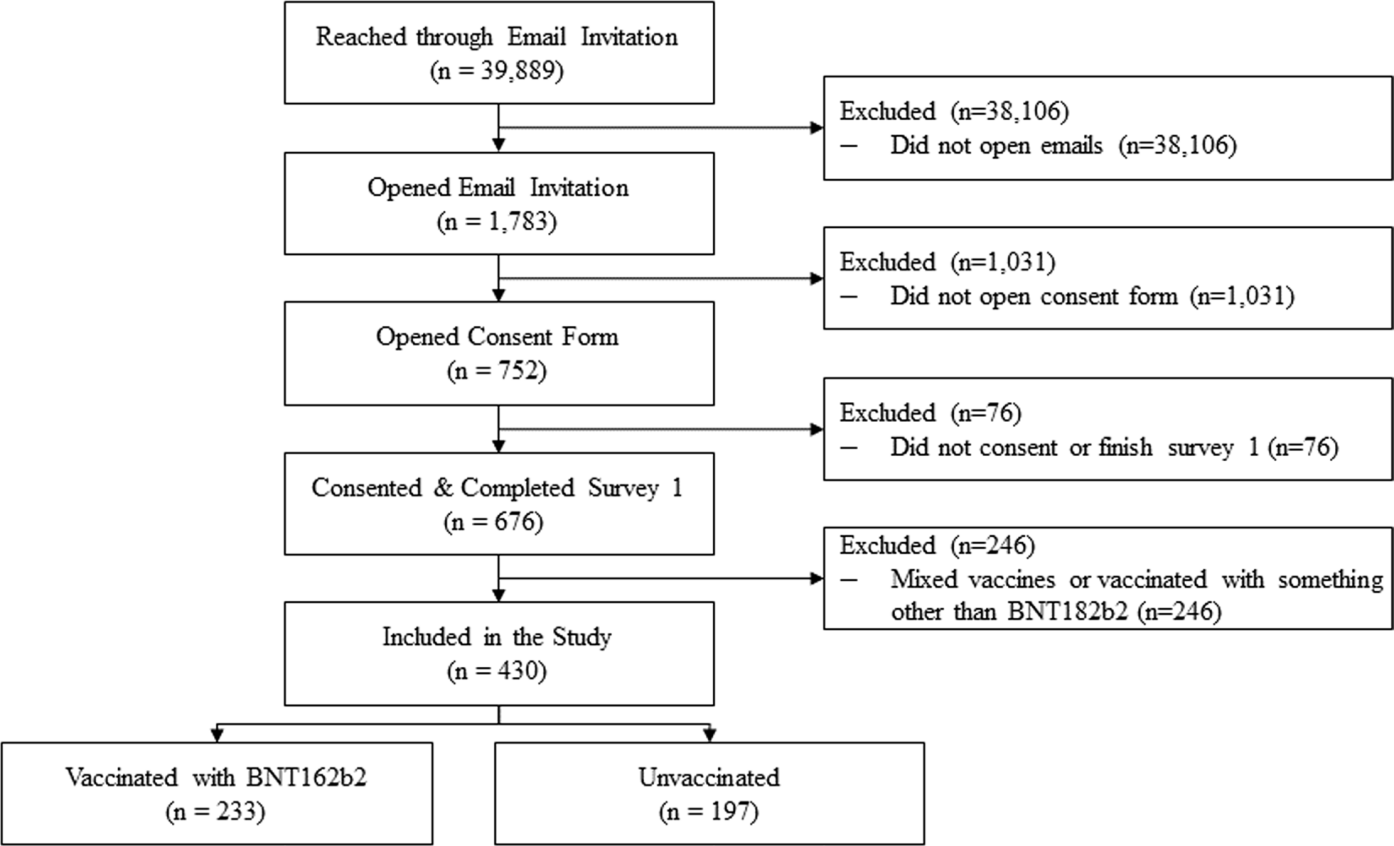
Study flow diagram

A total of 39,889 eligible candidates were outreached. Of those, 676 consented and completed the first survey, for a consent rate of 1.7%. Compared with individuals in the CVS Health analytic dataset who did not participate in our study, the study sample was over-represented by women and Caucasians, with slightly more individuals vaccinated and with comorbidities (Additional file 1: Table S1). The final study population included 430 subjects (Fig. 2). 100% completed the EQ-5D-5L questionnaire at pre-COVID-19 baseline and at Day 3, and 77.0% completed it at Week 4. The WPAI-GH questionnaire was completed by 88.1% of the participants at pre-COVID-19 baseline, 88.1% at Week 1 and 76.9% at Week 4.

The sociodemographic characteristics of the baseline participants are shown in Table 1. Overall, the mean (SD) age was 42.4 (14.3), 76% were female, 68.6% Caucasian, 58.7% from Southern US. There were 24.2% participants who reported ≥ 1 comorbidities, including 4.4% with immunocompromising conditions and 39% reported a previous COVID-19 infection.

**Table 1:** Patient characteristics on index day

	All	BNT162b2	Unvaccinated	*P* value ^a^
Total, n	430	233	197	
Age, years				
Mean, SD	42.4 (14.3)	43.7 (15.3)	40.9 (12.9)	0.049
18–29	87 (20.2%)	49 (21.0%)	38 (19.3%)	0.011
30–49	213 (49.5%)	100 (42.9%)	113 (57.4%)	
50–64	94 (21.9%)	60 (25.8%)	34 (17.3%)	
≥ 65	36 (8.4%)	24 (10.3%)	12 (6.1%)	
Gender				0.966
Female	327 (76.0%)	177 (76.0%)	150 (76.1%)	
Male	103 (24.0%)	56 (24.0%)	47 (23.9%)	
Race/ethnicity				0.026
White or Caucasian (not Hispanic or Latino)	295 (68.6%)	166 (71.2%)	129 (65.5%)	
Black or African American	20 (4.7%)	7 (3.0%)	13 (6.6%)	
Hispanic	61 (14.2%)	35 (15.0%)	26 (13.2%)	
Asian	22 (5.1%)	15 (6.4%)	7 (3.6%)	
Patient refused	13 (3.0%)	5 (2.2%)	8 (4.1%)	
Other	19 (4.4%)	5 (2.2%)	14 (7.1%)	
CMS geographic region (n, %)				0.009
Region 1: ME, NH, VT, MA, CT, RI	19 (4.4%)	10 (4.3%)	9 (4.6%)	
Region 2: NY, NJ, PR, VI	11 (2.6%)	7 (3.0%)	4 (2.0%)	
Region 3: PA, DE, MD, DC, WV, VA	37 (8.6%)	22 (9.4%)	15 (7.6%)	
Region 4: KY, TN, NC, SC, GA, MS, AL, FL	156 (36.3%)	81 (34.8%)	75 (38.1%)	
Region 5: MN, WI, IL, MI, IN, OH	58 (13.5%)	31 (13.3%)	27 (13.7%)	
Region 6: NM, OK, AR, TX, LA	82 (19.1%)	56 (24.0%)	26 (13.2%)	
Region 7: NE, IA, KS, MO	19 (4.4%)	11 (4.7%)	8 (4.1%)	
Region 8: MT, ND, SD, WY, UT, CO	1 (0.2%)	1 (0.4%)	0 (0.0%)	
Region 9: CA, NV, AZ, GU	46 (10.7%)	13 (5.6%)	33 (16.8%)	
Region 10: AK, WA, OR, ID	1 (0.2%)	1 (0.4%)	0 (0.0%)	
U.S. geographic region				0.005
Northeast	53 (12.2%)	29 (12.3%)	24 (12.2%)	
South	254 (58.7%)	150 (63.6%)	104 (52.8%)	
Midwest	77 (17.8%)	42 (17.8%)	35 (17.8%)	
West	49 (11.3%)	15 (6.4%)	34 (17.3%)	
Previously tested positive	167 (38.8%)	89 (38.2%)	78 (39.6%)	0.589
Work in healthcare	47 (10.9%)	29 (12.4%)	18 (9.1%)	0.309
Work in high-risk setting	44 (10.2%)	30 (12.9%)	14 (7.1%)	0.158
Live in high-risk setting	22 (5.1%)	12 (5.2%)	10 (5.1%)	0.553
Social vulnerability index, mean (SD)	0.44 (0.22)	0.40 (0.22)	0.49 (0.21)	< 0.001
Self-reported comorbidity				
Asthma or chronic lung disease	34 (7.9%)	21 (9.0%)	13 (6.6%)	0.355
Cirrhosis of the liver	1 (0.2%)	1 (0.4%)	0 (0.0%)	1.0000
Immunocompromised conditions or weakened immune system^c^	19 (4.4%)	12 (5.2%)	7 (3.6%)	0.422
Diabetes	20 (4.7%)	13 (5.6%)	7 (3.6%)	0.320
Heart conditions or hypertension	52 (12.1%)	30 (12.9%)	22 (11.2%)	0.588
Overweight or obesity	19 (4.4%)	12 (5.2%)	7 (3.6%)	0.422
At least 1 comorbidity	104 (24.2%)	61 (26.2%)	43 (21.8%)	0.294
Number of comorbidities, mean (SD)	0.34 (0.68)	0.38 (0.75)	0.28 (0.58)	0.138
*Index day*^b^ *acute COVID-19 symptoms*				
Systemic symptoms				
Fever	164 (38.1%)	71 (30.5%)	93 (47.2%)	< 0.001
Chills	213 (49.5%)	100 (42.9%)	113 (57.4%)	0.003
Muscle or body aches	232 (54.0%)	115 (49.4%)	117 (59.4%)	0.038
Headache	293 (68.1%)	153 (65.7%)	140 (71.1%)	0.231
Fatigue	266 (61.9%)	141 (60.5%)	125 (63.5%)	0.532
Respiratory symptoms				
Shortness of breath or difficulty breathing	54 (12.6%)	25 (10.7%)	29 (14.7%)	0.213
Cough	309 (71.9%)	168 (72.1%)	141 (71.6%)	0.903
Sore throat	238 (55.3%)	134 (57.5%)	104 (52.8%)	0.327
New/recent loss of taste or smell	45 (10.5%)	23 (9.9%)	22 (11.2%)	0.662
Congestion or runny nose	322 (74.9%)	188 (80.7%)	134 (68.0%)	0.003
GI symptoms				
Nausea or vomiting	55 (12.8%)	24 (10.3%)	31 (15.7%)	0.093
Diarrhea	88 (20.5%)	37 (15.9%)	51 (25.9%)	0.010
Number of acute COVID-19 symptoms, mean (SD)	5.3 (2.6)	5.1 (2.4)	5.6 (2.7)	0.034

^a^*P* value refers to the comparison between BNT162B2 and Unvaccinated

^b^COVID-19 test nasal swab day

^c^Immunocompromised conditions includes compromised immune system (such as from immuno-compromising drugs, solid organ or blood stem cell transplant, HIV, or other conditions), conditions that result in a weakened immune system, including cancer treatment, and kidney failure or end stage renal disease

About 46% (197) were unvaccinated and 54% (233) were vaccinated with BNT162b2; of those, respectively 140 (60%) and 93 (40%) received 2 and 3 doses. Compared with unvaccinated, BNT162b2 participants were comparable with respect to gender, working and living settings, and comorbidities. Vaccinated participants wereslightly older with mean age 43.7 vs. 40.9 (*p* = 0.049); reported living in a less vulnerable area with lower mean social vulnerability index (0.40 vs. 0.49, *P* <  < 0.001); and reported slight differences in race/ethnicity and region. In the vaccinated group, mean (SD) time since vaccination before infection was 186 (105) days.

At index date, the most reported acute symptoms were respiratory and systemic. BNT162b2 vaccinated participants reported fewer overall acute COVID-19 symptoms on average than unvaccinated participants, mean 5.1 vs. 5.6, *P* = 0.034 (Table 1). Directionally, the proportions of all systemic and GI-related symptoms were numerically lower in the BNT162b2 cohort. Relative to unvaccinated, those vaccinated with BNT162b2 reported significantly fewer symptoms of fever (30.5% vs. 47.2% *P* < 0.001), chills (42.9% vs. 57.4%, *P* = 0.003), muscle or body aches (49.4% vs. 59.4%, *P* = 0.038), and diarrhea (15.9% vs. 25.9%, *P* = 0.010), but more congestion or runny nose (80.7% vs. 68.0%, *P* = 0.003).

### Post-COVID-19 symptoms

At Week 4, the mean number of symptoms was statistically lower in the BNT162B2 cohort (2.5 vs. 3.7, *p* = 0.002). The overall prevalence also decreased over time, specifically fever, cough, headache, fatigue, diarrhea, muscle pain; however, ~ 70% of participants still reported at least 1 post-COVID-19 symptom. Directionally, the proportions of all symptoms were numerically lower in the BNT162b2 cohort. Symptoms of worsening after physical or mental activities (10.3% vs. 20.6%), general pain/discomfort (11.4% vs. 19.4%), change in smell or taste (10.9% vs. 20.6%), headache (16.0% vs. 25.2%), sleep problems (20.0% vs. 29.7%), mood changes (7.4% vs. 14.8%), memory loss (6.3% vs. 17.4%) and diarrhea (3.4% vs. 11.0%) were statistically significant (*P* < 0.05) (Table 2).

**Table 2:** Post-COVID-19 symptoms at week 4

Symptom	All	BNT162b2	Unvaccinated	*P* value^a^
General symptoms				
Tiredness or fatigue	136 (41.2%)	71 (40.6%)	65 (41.9%)	0.802
Symptoms that get worse after physical or mental ctivities	50 (15.2%)	18 (10.3%)	32 (20.6%)	0.009
Fever	1 (0.3%)	0 (0.0%)	1 (0.7%)	0.470
General pain/discomfort	50 (15.2%)	20 (11.4%)	30 (19.4%)	0.045
Respiratory and cardiac				
Difficulty breathing or shortness of breath	58 (17.6%)	26 (14.9%)	32 (20.6%)	0.168
Cough	86 (26.1%)	40 (22.9%)	46 (29.7%)	0.159
Chest or stomach pain	32 (9.7%)	14 (8.0%)	18 (11.6%)	0.268
Fast-beating or pounding heart (also known as heart palpitations)	38 (11.5%)	17 (9.7%)	21 (13.5%)	0.276
Neurologic				
Change in smell or taste	51 (15.5%)	19 (10.9%)	32 (20.6%)	0.014
Headache	67 (20.3%)	28 (16.0%)	39 (25.2%)	0.039
Dizziness on standing (lightheadedness)	45 (13.6%)	20 (11.4%)	25 (16.1%)	0.214
Difficulty thinking or concentrating (sometimes referred to as “brain fog”)	86 (26.1%)	43 (24.6%)	43 (27.7%)	0.513
Pins-and-needles feeling	24 (7.3%)	10 (5.7%)	14 (9.0%)	0.247
Sleep problems	81 (24.5%)	35 (20.0%)	46 (29.7%)	0.042
Mood changes	36 (10.9%)	13 (7.4%)	23 (14.8%)	0.031
Memory loss	38 (11.5%)	11 (6.3%)	27 (17.4%)	0.002
Other				
Diarrhea	23 (7.0%)	6 (3.4%)	17 (11.0%)	0.007
Joint or muscle pain	67 (20.3%)	29 (16.6%)	38 (24.5%)	0.073
Rash	11 (3.3%)	3 (1.7%)	8 (5.2%)	0.082
Changes in period cycles	28 (11.5%)	12 (9.4%)	16 (13.8%)	0.280
Number of post-COVID-19 symptoms, mean (SD)	3.1 (3.6)	2.5 (3.0)	3.7 (4.1)	0.002
0	100 (30.3%)	54 (30.9%)	46 (29.7%)	0.001
1–2	100 (30.3%)	59 (33.7%)	41 (26.5%)	
3–5	61 (18.5%)	40 (22.9%)	21 (13.5%)	
6–8	32 (9.7%)	9 (5.1%)	23 (14.8%)	
≥ 9	37 (11.2%)	13 (7.4%)	24 (15.5%)	

^a^P-values of t-test for number of symptoms, chi-square or Fisher’s exact tests when any one cell has an expected frequency less than 5 for individual symptoms and number of symptom category comparing the BNT162b2 cohort and the unvaccinated cohort

### Health-related quality of life

#### Utility index scores

Mean pre-COVID-19 baseline UIs did not differ between the BNT162b2 and unvaccinated cohorts, respectively (0.924 and 0.918, *P* = 0.547). COVID-19 infection had a detrimental effect on the HRQoL of participants, especially during the acute episode (Day 3). In both the BNT162b2 and the unvaccinated cohorts, UIs were lower at Day 3 and Week 4 relative to pre-COVID-19. While UI improvement was observed over time, the UI did not return to pre-COVID levels at Week 4 (Table 3).

**Table 3:** Summary of EQ-5D-5L and WPAI-GH Scores^a^ and their changes from baseline by assessment time

	BNT162b2 cohort						Unvaccinated cohort						Difference in change from baseline between cohorts		
	Score		Change from baseline^b^				Score		Change from baseline						
	n	Mean (SD)	n	Mean (SD)	*P* value^c^	ES_w_^d^	n	Mean (SD)	n	Mean (SD)	*P* value^c^	ES_w_^d^	Mean (SD)	*P* value^e^	ES_b_^f^
*EQ-5D-5L*															
Visual analogue scale (VAS)															
Baseline^g^	233	86.9 (10.7)					197	87.8 (11.0)						0.414 ^h^	
Day 3	233	73.9 (15.6)	233	− 13.0 (12.5)	< 0.001	− 1.04	194	71.8 (19.6)	194	− 16.1 (17.1)	< 0.001	− 0.94	3.1 (14.8)	0.033	0.21
Week 4	171	82.9 (13.8)	171	− 3.7 (10.7)	< 0.001	− 0.35	151	80.9 (15.7)	151	− 7.2 (13.7)	< 0.001	− 0.53	3.5 (12.2)	0.011	0.28
Utility Index (US weight)															
Baseline^e^	233	0.924 (0.117)					197	0.918 (0.117)						0.547 ^h^	
Day 3	233	0.820 (0.193)	233	− 0.105 (0.159)	< 0.001	− 0.66	197	0.762 (0.252)	197	− 0.155 (0.228)	< 0.001	− 0.68	0.050 (0.194)	0.007	0.26
Week 4	176	0.882 (0.145)	176	− 0.039 (0.120)	< 0.001	− 0.33	155	0.838 (0.197)	155	− 0.074 (0.156)	< 0.001	− 0.47	0.035 (0.138)	0.023	0.25
*WPAI-GH*															
Absenteeism															
Baseline^e^	153	2.8 (11.8)					129	11.7 (26.4)						< 0.001 ^h^	
Week 1	153	44.9 (38.3)	147	42.5 (38.8)	< 0.001	1.09	129	66.7 (37.0)	127	55.5 (39.2)	< 0.001	1.42	− 13.0 (39.0)	0.006	− 0.33
Week 4	128	4.6 (17.2)	122	1.4 (20.1)	0.460	0.07	106	3.3 (12.2)	102	− 7.7 (28.1)	0.007	− 0.27	9.0 (24.1)	0.006	0.37
Presenteeism															
Baseline^e^	153	9.5 (18.5)					124	9.4 (20.4)						0.958 ^h^	
Week 1	123	41.7 (27.4)	119	33.0 (29.1)	< 0.001	1.13	82	47.8 (34.2)	81	38.2 (35.9)	< 0.001	1.06	− 5.1 (32.1)	0.268	− 0.16
Week 4	125	11.7 (18.8)	119	1.4 (24.5)	0.527	0.06	105	18.7 (23.4)	96	10.7 (24.1)	< 0.001	0.45	− 9.3 (24.3)	0.006	− 0.38
Work productivity loss															
Baseline^e^	152	10.5 (20.0)					123	14.2 (24.7)						0.175 ^h^	
Week 1	123	56.6 (31.5)	118	46.7 (34.5)	< 0.001	1.35	82	66.9 (32.4)	80	53.2 (35.3)	< 0.001	1.51	− 6.4 (34.9)	0.203	− 0.18
Week 4	125	12.9 (20.7)	118	1.4 (27.5)	0.589	0.05	105	19.8 (24.6)	96	7.7 (30.6)	0.016	0.25	− 6.3 (29.0)	0.113	− 0.22
Activity impairment															
Baseline^e^	203	12.5 (22.2)					176	17.2 (27.2)						0.065 ^h^	
Week 1	202	48.6 (29.8)	202	36.1 (31.9)	< 0.001	1.13	176	54.2 (32.7)	176	37.0 (37.5)	< 0.001	0.99	− 0.9 (34.6)	0.801	− 0.03
Week 4	175	15.5 (21.7)	175	2.3 (26.3)	0.242	0.09	155	25.9 (28.5)	155	8.8 (33.8)	0.002	0.26	− 6.4 (30.1)	0.053	− 0.21

^a^Score ranges: EQ-5D-5L VAS 0 to 100, EQ-5D-5L UI (the United States weights)  − 0.573 to 1; WPAI-GH (absenteeism, presenteeism, work productivity loss, and activity impairment) 0 to 100 percent

^b^Baseline refers to pre-COVID-19 symptom onset

^c^*P* value of t-test comparing mean score changes from baseline and 0 within BNT162b2 or Unvaccinated cohorts

^d^ES_w_ refers to effect size for score changes from baseline within BNT162b2 or Unvaccinated cohorts

^e^*P* value of t-test comparing mean score changes from baseline between BNT162b2 and Unvaccinated cohorts

^f^ES_b_ refers to effect size for score changes from baseline between BNT162b2 and Unvaccinated cohorts

^g^Prior to symptom onset (pre-COVID)

^h^*P* values of t-tests comparing pre-COVID-19 baseline mean scores between BNT162b2 and Unvaccinated cohorts

The BNT162B2 cohort was less impacted than the unvaccinated cohort, at both Day 3 and Week 4. After controlling for pre-COVID baseline score and other covariates, the least-square estimate UI scores at Day 3 were 0.77 and 0.84 in the unvaccinated and BNT162B2 cohorts, respectively (Table 4). Moderate ESs of 0.64 and 0.49 were observed from baseline. At Week 4, the least-square estimate UI scores were 0.86 and 0.90. Small-to-moderate ESs of 0.38 and 0.13 were observed from baseline, respectively. The differences between the two groups were statistically significant (*P* < 0.05). (Table 4) Small-to-medium ESs between cohorts were observed and were 0.36 and 0.32 for Day 3 and Week 4, respectively. (Table 4, Additional file 2: Figs. S1 and S2).

**Table 4:** Least-square mean estimate and 95% confidence interval for EQ-5D-5L and WPAI-GH scores^a^

	BNT162b2 cohort				Unvaccinated cohort				Between cohort difference		
	Score	Change from baseline			Score	Change from baseline					
Variable	LSE (95% CI)	LSE (95% CI)	*P* value^b^	ES_w_^c^	LSE (95% CI)	LSE (95% CI)	*P* value^b^	ES_w_^c^	LSE (95% CI)	*P* value^d^	ES_b_^e^
*EQ-5D-5L*											
Visual analogue scale (VAS)											
Day 3	76.2 (73.3, 79.2)	− 11.1 ( − 14.1, − 8.2)	< 0.001	− 0.89	72.6 (69.7, 75.4)	− 14.8 ( − 17.6, − 11.9)	< 0.001	− 0.86	3.6 (0.8, 6.5)	0.013	0.25
Week 4	85.0 (82.1, 87.9)	− 2.3 ( − 5.2, 0.6)	0.119	− 0.22	81.6 (78.8, 84.4)	− 5.7 ( − 8.5, − 2.9)	< 0.001	− 0.42	3.4 (0.6, 6.2)	0.016	0.28
Utility Index (US weight)											
Day 3	0.842 (0.805, 0.879)	− 0.077 ( − 0.115, − 0.040)	< 0.001	− 0.49	0.773 (0.736, 0.809)	− 0.147 ( − 0.183, − 0.110)	< 0.001	− 0.64	0.069 (0.032, 0.107)	< 0.001	0.36
Week 4	0.903 (0.868, 0.938)	− 0.016 ( − 0.051, 0.019)	0.369	− 0.13	0.859 (0.826, 0.892)	− 0.060 ( − 0.093, − 0.027)	< 0.001	− 0.38	0.044 (0.013, 0.075)	0.005	0.32
*WPAI-GH*											
Absenteeism											
Week 1	45.6 (38.0, 53.1)	39.1 (31.6, 46.7)	< 0.001	1.01	65.0 (57.5, 72.5)	58.6 (51.1, 66.0)	< 0.001	1.49	− 19.4 ( − 28.3, − 10.6)	< 0.001	− 0.50
Week 4	5.3 (0.0, 10.7)	− 1.1 ( − 6.5, 4.2)	0.676	− 0.06	2.2 (0.0, 7.1) ^f^	− 4.3 ( − 9.2, 0.6)	0.086	− 0.15	3.2 ( − 1.1, 7.4)	0.145	0.13
Presenteeism											
Week 1	38.4 (30.3, 46.5)	29.0 (20.8, 37.1)	< 0.001	1.00	46.8 (38.6, 55.1)	37.4 (29.1, 45.6)	< 0.001	1.04	− 8.4 ( − 16.7, − 0.1)	0.047	− 0.26
Week 4	6.8 (0, 13.9)^f^	− 2.7 ( − 9.7, 4.4)	0.458	− 0.11	16.0 (9.4, 22.5)	6.5 (0.0, 13.0)	0.050	0.27	− 9.2 ( − 14.7, − 3.6)	0.001	− 0.38
Work productivity loss											
Week 1	53.8 (45.1, 62.4)	42.4 (33.8, 51.0)	< 0.001	1.23	65.0 (56.2, 73.7)	53.6 (44.8, 62.4)	< 0.001	1.52	− 11.2 ( − 19.9, − 2.5)	0.012	− 0.32
Week 4	8.6 (0.9, 16.2)	− 2.8 ( − 10.4, 4.9)	0.476	− 0.10	17.0 (9.9, 24.0)	5.7 ( − 1.4, 12.7)	0.116	0.18	− 8.4 ( − 14.5, − 2.3)	0.007	− 0.29
Activity impairment											
Week 1	43.9 (37.7, 50.1)	29.0 (22.8, 35.3)	< 0.001	0.91	50.2 (44.2, 56.2)	35.4 (29.3, 41.4)	< 0.001	0.94	− 6.3 ( − 12.4, − 0.2)	0.044	− 0.18
Week 4	11.0 (5.1, 16.9)	− 3.9 ( − 9.7, 2.0)	0.194	− 0.15	21.3 (15.7, 26.9)	6.4 (0.8, 12.0)	0.025	0.19	− 10.3 ( − 15.6, − 5.0)	< 0.001	− 0.34

*LSE* Least-Square Mean Estimate, *CI* Confidence Interval

^a^Multivariate models include variables for time, vaccination status and interaction of time by vaccination status, as well as covariates of participant pre-COVID-19 symptom onset score, sociodemographic characteristics (age, sex, regions, social vulnerability, race/ethnicity, high risk occupations), previously tested positive for COVID-19, severity of acute illness (number of symptoms reported on index date), and immunocompromised status. Parameter estimates are presented in Additional file 1: Table S3

^b^*P* value refers to the comparison of lease-square mean estimates score changes from baseline and 0 within BNT162b2 or Unvaccinated cohorts

^c^ES_w_, within-cohort effect size, was calculated as the least square estimate of mean change from divided by the observed standard deviation of change scores from baseline to follow-up

^d^ES_b_, between-cohort effect size, was calculated as the difference in least square estimates of mean changes from baseline between cohorts, divided by the observed pooled standard deviation of change scores

^e^*P* value refers to the difference in lease-square mean estimates between BNT162b2 and Unvaccinated cohorts

^f^Lower limit of 95% CI was truncated from  − 2.7 to 0 for absenteeism and  − 0.3 to 0 for presenteeism at Week 4 because the valid range is 0–100

#### EQ-VAS

The pattern of EQ-VAS scores was similar to that observed for UI. Mean pre-COVID-19 baseline EQ-VAS scores were similar for the BNT162b2 and unvaccinated cohorts, respectively 86.9 and 87.8 (*P* = 0.414) (Table 3). Similar to the UIs, the pre-COVID EQ-VAS were rated relatively high by the participants, indicating a generally healthy cohort. The least-square estimate EQ-VAS scores for the BNT162b2 and unvaccinated cohorts were, respectively, 76.2 and 72.6 at Day 3 and 85.0 and 81.6 at Week 4. After controlling for pre-COVID-19 baseline score and other covariates, the least-square estimates of change from pre-COVID-19 baseline in EQ VAS for the BNT162B2 and the unvaccinated cohort were − 11.1 and − 14.8, respectively on Day 3, and − 2.3 and -5.7, respectively at Week 4. COVID-19 had a large adverse impact on EQ-VAS with an ES of − 0.89 for BNT162B2 cohort and − 0.86 for Unvaccinated cohort on Day 3, and small ES ( − 0.22) for BNT162B2 cohort and approaching medium ES ( − 0.42) for Unvaccinated cohort at Week 4. BNT162B2 cohort was associated with 3.6 (*P* = 0.013) on Day 3 and 3.4 (*P* = 0.016) at Week 4 less drop in EQ VAS than the Unvaccinated cohort. The ESs between cohorts were small yet relevant; 0.25 and 0.28 for Day 3 and Week 4, respectively (Table 4, Additional file 2: Figs. S1 and S2).

#### EQ-5D-5L dimensions

**Fig. 3:**
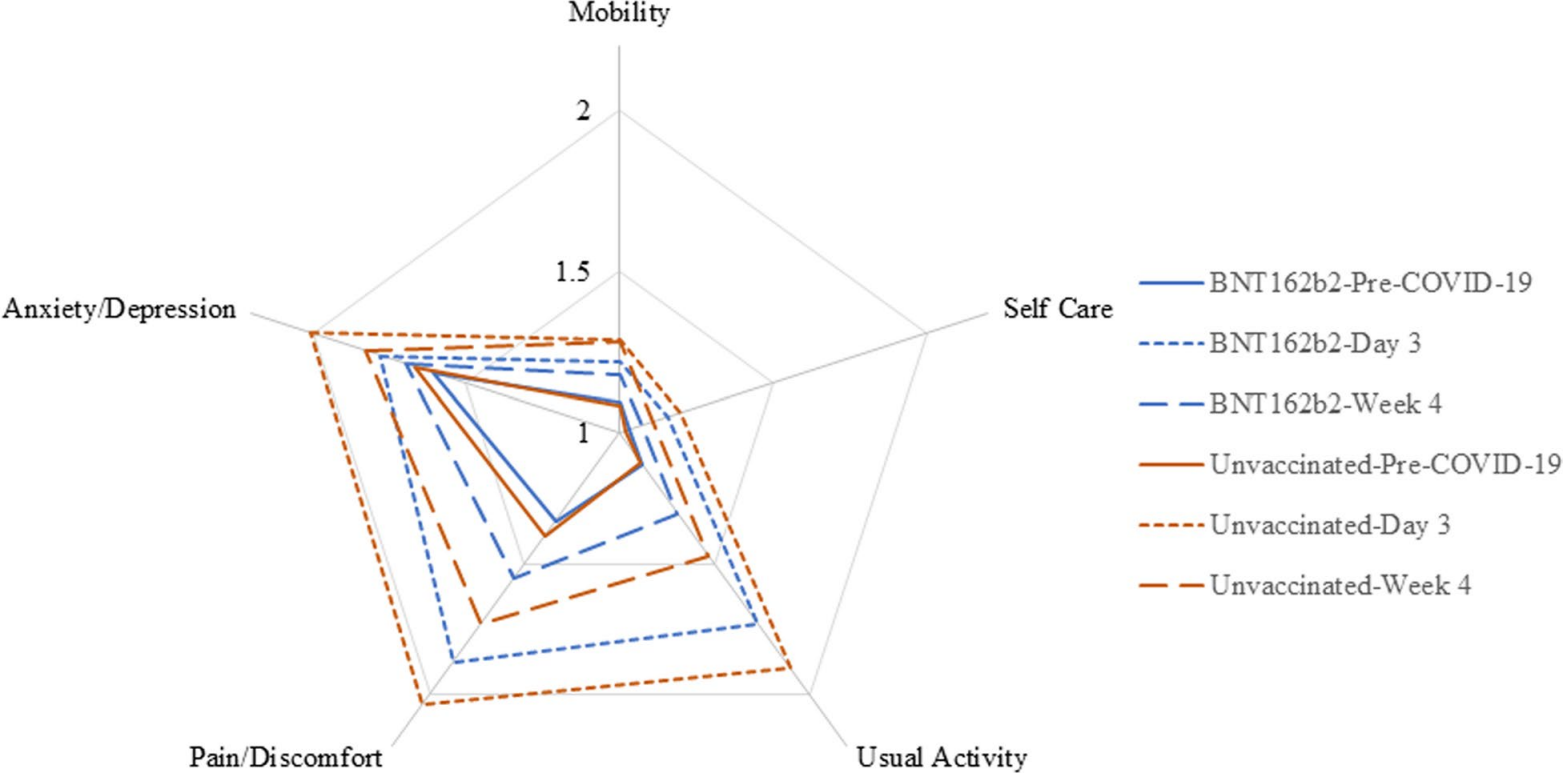
Mean Responses of EQ-5D-5L Dimensions by Timepoint. Mean dimension scores range from 1 for no problem to 5 for extreme/unable. The blue and red solid lines indicate that vaccinated and unvaccinated were similar at the pre-COVID baseline. At Day 3 and Week 4 post-index date, vaccinated cohort was less impacted (lower scores) than unvaccinated by COVID on anxiety/depression, pain/discomfort, and usual activities (dotted lines for Day 3, dashed lines for Week 4)

The health status of the study participants according to the dimensions of EQ-5D-5L is reported in Fig. 3 and Additional file 1: Table S2. In both groups, at Day 3, over half of the cohort reported problems in usual activities, pain/discomfort and anxiety/depression, while the vast majority reported no or slight problems in mobility and self-care. At Week 4, the vast majority continued to report no or slight problems with mobility, self-care, as well as for usual activities; most reported no, slight or moderate problems with pain/discomfort and anxiety/depression. The BNT162b2 cohort had lower mean responses across all 5 domains at both Day 3 and Week 4 relative to unvaccinated.

### Work productivity and activity impairment

Approximately 65% of participants reported being currently employed at baseline (155 in the BNT162b2 cohort and 129 unvaccinated), and were eligible to complete the absenteeism, presenteeism and work-productivity loss questions. At Week 1, COVID-19 had a large impact on all four WPAI-GH domains for both the unvaccinated and BNT162b2cohort. The mean time loss due to absenteeism was, respectively, 65.0% and 45.6%; the mean time loss due to presenteeism was, respectively, 46.8% and 38.4%; the mean time of work productivity loss was 65.0% and 53.8%, and the mean time of activity impairment was 50.2% and 43.9%. All within-cohort ESs were > 0.8, which are considered large effects (Table 3). After controlling for pre-COVID-19 baseline score, and other covariates, the BNT162b2 cohort was associated with less worsening in WPAI-GH scores. Small-to-medium ESs were observed for work-related scores (absenteeism -0.50, presenteeism -0.26, and work productivity loss -0.32) between the BNT162b2 cohort and the unvaccinated cohort (Table 4). At Week 4, the mean time loss dropped across all four domains. The time loss due to absenteeism dropped substantially; the change from baseline in absenteeism was not found to be statistically significant between the BNT162b2 cohort and the unvaccinated cohort. Small-to-medium ESs were observed for presenteeism (-0.38) work productivity loss (-0.29), and activity impairment (-0.34) between the BNT162b2 cohort and the unvaccinated cohort (Table 4, Additional file 2: Figs. S3 and S4).

## Discussion

The impacts of SARS-CoV-2 infection go beyond its clinical outcomes. In this study, shortly after infection, the UI and EQ-VAS HRQoL scores dropped from pre-COVID, and over half of the study population reported problems in usual activities, pain/discomfort and anxiety/depression. At Week 1, the work productivity and activity impairment time loss were over 50%. At Week 4, both the HRQoL and WPAI scores improved, although they did not return to pre-COVID levels. Individuals vaccinated with BNT162b2 were less impacted and recovered faster than unvaccinated individuals. Multivariable analyses showed that BNT162b2 was significantly associated with higher EQ-VAS and UI scores, and less symptoms and better WPAI scores, except for absenteeism at Week 4. These results indicate an additive benefit beyond vaccine effectiveness that should be explored further.

To our knowledge, this is the first study measuring the impact of COVID-19 on the HRQoL and WPAI among outpatients. In contrast to our study, previous research that used EQ-5D scales to measure COVID-19 impact on HRQoL reported mean UI scores ranging from 0.61 to 0.86 depending on the hospitalization treatment and time since discharge [1, 2]. The EQ-VAS scores ranged from 50.7 to 70.3 [1, 2]. In our study, the HRQoL scores at Day 3 and Week 4 are higher than those, likely due to the different study populations and periods. In a small US study assessing the impact of COVID-19 on WPAI ~ 4 months post-infection among subjects enrolled in clinical trials before the introduction of vaccines, 46% of the non-hospitalized patients reported impairment in daily activities [26]. Among the employed, 11.5% missed work and 38.9% reported impairment at work due to health. In our study, all the WPAI scores among unvaccinated at Week 1 are higher, and those at Week 4 similar or lower than those, likely due to the different cut-off, study populations, periods and design.

Strengths of this study include the nationwide real-world source population of mildly symptomatic outpatients, the prospective collection of primary outcomes via validated instruments, and the representativeness of the employed population for work productivity analyses. The age distribution of study participants was comparable with the non-enrolled tested population (*p* = 0.076 for mean age, Additional file 1: Table S1) and with CDC research in non-hospitalized adults. [27]

The study is subject to limitations. All the data analyzed was self-reported and may be subject to error, missingness, recall bias, social desirability bias, and selection bias associated with survey drop-out. Out of 430 participants completing Day 3 survey, 12% (51/430) missed Week 1 and 23% (99/430) missed Week 4 survey. The drop in responses may partly be the result of responders’ fatigue, and/or recovered cases not returning to follow-up surveys (Additional file 1: Table S4).

The study population differed from non-enrolled tested outpatients. The female over-representation is in line with prior research indicating that women are more likely to contribute to health research surveys [2]. Various models were fit to account for potential effects due to sociodemographic factors and comorbidities. These adjusted ESs between BNT162b2 and unvaccinated cohorts were similar and consistent with those calculated from observed data (unadjusted ESs).

The pre-COVID baseline scores were slightly higher than US population norms [28]. The healthy pre-infection status of the study population and the potential for retrospective recall bias may partially explain the difference.

The pre-COVID-19 values for absenteeism and presenteeism were 3.1% and 9.5% in the BNT162b2 cohort, and were generally in line with Tundia et al. (2015) [29], whom reported 4% absenteeism and 10% presenteeism for the US population. The reported values were slightly higher among unvaccinated, 11.7% and 9.4% respectively.

There is currently no standard definition of minimal clinically important difference of PROs in COVID-19 research. We used ES of Cohen’s d to quantify the magnitude of score change from baseline within the BNT162b2 vaccinated cohort and the unvaccinated cohort, as well the difference between these two cohorts [22]. An ES of 0 between groups indicates that the average (typical) vaccinated person has a score that is no different from the typical control person; equivalently, scores of the typical vaccinated person are more favorable than 50% of the individual scores in the control group, meaning no incremental benefit. If the vaccinated cohort is presumed more effective than the unvaccinated cohort, ES thresholds of 0.2, 0.3, 0.5, and 0.8 (in absolute value) indicate that, based on the standardized normal distribution, the score of the typical person in the vaccinated cohort is more favorable than 58% (8% incremental benefit), 62% (12% incremental benefit), 69% (19% incremental benefit), and 70% (29% increment benefit) of the scores from individuals in the unvaccinated cohort. For example, from baseline to Week 1, the increase in the absenteeism WPAI score of the typical person in the vaccinated cohort was less (more favorable) than the corresponding change in 69% of individuals in the unvaccinated group (effect size =  − 0.5). Depending on the type of outcome, the same type of effect size interpretation for between cohorts can be given within cohort.

The study did not assess the impact on pediatrics, caregivers, long-term outcomes (e.g., “Long COVID”), and the data was collected during Omicron predominance in the US. Therefore, these findings may not be generalizable to prior or future variants, other countries, time periods and populations that were excluded. COVID-19 sequalae can affect a substantial portion of patients, with long-term consequences for their health, continuity of care and ability to work [1, 2]. Persistent symptoms and work impairment were reported ~ 4 months after infection among non-hospitalized US patients enrolled in clinical trials [26]. Continued follow-up studies covering longer time periods may inform whether the protection provided by COVID-19 vaccination extends beyond the acute phase. Only generic validated PROMs were used in this study; COVID-19 disease-specific instruments are under development [30, 31], warranting research on their implementation. The PROMs omitted questions on vaccine adverse events. The mean 6-month interval between vaccination and breakthrough infection and a medical review ruled out cases of residual symptoms from vaccination. Research on the impact of vaccine adverse events on HRQoL is warranted.

Lastly, the study adopted an observational design, which is limited in establishing causal relationships. Future studies using different data collection methods could corroborate the study findings.

## Conclusion

This study found that mild COVID-19 infection at a time of Omicron predominance adversely impacted the HRQoL, daily activity and work productivity of patients. This detrimental effect improved over time, although it persisted for at least one month post infection. Compared with unvaccinated, those vaccinated with BNT162b2 were less impacted and recovered faster. These findings advance research on COVID-19 associated humanistic outcomes and the potential effect of BNT162b2 in lessening the loss of HRQoL, daily activity and work productivity due to COVID-19. The results can inform the estimation of quality-adjusted life years and indirect cost savings in health economic studies.

## Supplementary Information

**Additional file 1: Table S1. **Patient Characteristics by Enrollment Status. **Table S2. **Summary of EQ-5D-5L Dimensions. **Table S3.** Mixed Models for Repeated Measurements EQ-5D-5L and WPAI-GH Scores. **Table S4. **Model Predicting the Missingness at Week 1 and Week 4.

**Additional file 2** **Supplemental Figure 1** Least-Square Estimates and 95% Confidence Intervals of EQ-5D-5L Scores. **Supplemental Figure 2** Summary Results of EQ-5D-5L scores across time periods. **Supplemental Figure 3** Least-Square Estimates and 95% Confidence Intervals of WPAI-GH Scores. **Supplemental Figure 4** Summary Results of WPAI-GH scores across time periods.

## Data Availability

Aggregated data that support the findings of this study are available upon reasonable request from the corresponding author MDF, subject to review. These data are not publicly available due to them containing information that could compromise research participant privacy/consent.

## Abbreviations

CDC: Centers for disease control and prevention
CI: Confidence interval
EQ-5D-5L: EuroQoL group 5 dimension and 5 level questionnaire
ES: Effect size
GH: General health
HRQoL: Health related quality of life
MMRM: Mixed models for repeated measures
SD: Standard deviation
SE: Standard error
STROBE: The Strengthening the reporting of observational studies in epidemiology
UI: Utility index
VAS: Visual analogue scale
WPAI: Work productivity and impairment
