# Supplementary material for: Impact of COVID-19 and effects of BNT162b2 on patient-reported outcomes: quality of life, symptoms, and work productivity among US adult outpatients
Source: J Patient Rep Outcomes. 2022 Dec 5;6:123. doi: 10.1186/s41687-022-00528-w (PMC9722994; doi:10.1186/s41687-022-00528-w)
Supplement: Supplementary file 1 — Additional file 1: Table S1. Patient Characteristics by Enrollment Status. Table S2. Summary of EQ-5D-5L Dimensions. Table S3. Mixed Models for Repeated Measurements EQ-5D-5L and WPAI-GH Scores. Table S4. Model Predicting the Missingness at Week 1 and Week 4. [file 41687_2022_528_MOESM1_ESM.docx]

Supplemental Table 1 Patient Characteristics by Enrollment Status

| Patient characteristics | | Enrolled N=676 | Not-Enrolled  N=39,213 | P value |
| --- | --- | --- | --- | --- |
| Vaccinated | |  |  | <0.001 |
| Yes | | 465 (68.8%) | 22,125 (56.4%) |  |
| No | | 211 (31.2%) | 17,087 (43.6%) |  |
| Missing | |  |  |  |
| Age, years, Mean, SD | | 43.2 (14.7) | 42.1 (15.5) | 0.076 |
| Age Category |  |  |  | |
| 18-29 | 134 (19.8%) | 10,095 (25.7%) | 0.005 | |
| 30-49 | 317 (46.9%) | 16,706 (42.6%) |  | |
| 50-64 | 156 (23.1%) | 8,561 (21.8%) |  | |
| ≥65 | 69 (10.2%) | 3,850 (9.8%) |  | |
| Gender, n (%) | |  |  | <0.001 |
| Male | | 181 (26.8%) | 17,843 (45.5%) |  |
| Female | | 495 (73.2%) | 21,370 (54.5%) |  |
| Race / Ethnicity, n (%) | |  |  | <0.001 |
| White or Caucasian (non-Hispanic or Latino) | | 486 (71.9%) | 22,647 (57.8%) |  |
| Black or African American | | 32 (4.7%) | 4,162 (10.6%) |  |
| Hispanic or Latino | | 85 (12.6%) | 6,878 (17.5%) |  |
| Asian | | 35 (5.2%) | 2,551 (6.5%) |  |
| Patient Refused | | 16 (2.4%) | 1,535 (3.9%) |  |
| Other | | 22 (3.3%) | 1,439 (3.7%) |  |
| US Geographic Region | |  |  | 0.258 |
| Northeast | | 92 (13.6%) | 4,387 (11.2%) |  |
| South | | 402 (59.5%) | 24,305 (62.0%) |  |
| Midwest | 118 (17.5%) | 6,414 (16.4%) |  | |
| West | | 64 (9.5%) | 4,105 (10.5%) |  |
| Number of acute COVID-19 symptoms, Mean (SD) | | 5.2 (2.5) | 5.1 (2.5) | 0.732 |
| Number of comorbidities, Mean (SD) | | 0.3 (0.7) | 0.3 (0.6) | 0.021 |
| ≥1 comorbidity | 164 (24.3%) | 7,677 (19.6%) | 0.002 | |

Supplemental Table 2 Summary of EQ-5D-5L Dimensions

| Dimension | All | BNT162b2 | Unvaccinated | P-value ^a^ |
| --- | --- | --- | --- | --- |
| Pre-COVID-19 Baseline |  |  |  |  |
| Mobility |  |  |  | 0.841 |
| No problems | 396 (92.1%) | 213 (91.4%) | 183 (92.9%) |  |
| Slight problems | 30 (7.0%) | 18 (7.7%) | 12 (6.1%) |  |
| Moderate problems | 4 (0.9%) | 2 (0.9%) | 2 (1.0%) |  |
| Severe problems | 0 (0.0%) | 0 (0.0%) | 0 (0.0%) |  |
| Unable | 0 (0.0%) | 0 (0.0%) | 0 (0.0%) |  |
| Missing | 0 (0.0%) | 0 (0.0%) | 0 (0.0%) |  |
| Self-Care |  |  |  | 0.560 |
| No problems | 419 (97.4%) | 226 (97.0%) | 193 (98.0%) |  |
| Slight problems | 11 (2.6%) | 7 (3.0%) | 4 (2.0%) |  |
| Moderate problems | 0 (0.0%) | 0 (0.0%) | 0 (0.0%) |  |
| Severe problems | 0 (0.0%) | 0 (0.0%) | 0 (0.0%) |  |
| Unable | 0 (0.0%) | 0 (0.0%) | 0 (0.0%) |  |
| Missing | 0 (0.0%) | 0 (0.0%) | 0 (0.0%) |  |
| Usual Activities |  |  |  | 0.904 |
| No problems | 394 (91.6%) | 214 (91.8%) | 180 (91.4%) |  |
| Slight problems | 23 (5.4%) | 11 (4.7%) | 12 (6.1%) |  |
| Moderate problems | 12 (2.8%) | 7 (3.0%) | 5 (2.5%) |  |
| Severe problems | 1 (0.2%) | 1 (0.4%) | 0 (0.0%) |  |
| Unable | 0 (0.0%) | 0 (0.0%) | 0 (0.0%) |  |
| Missing | 0 (0.0%) | 0 (0.0%) | 0 (0.0%) |  |
| Pain / Discomfort |  |  |  | 0.614 |
| No | 307 (71.4%) | 172 (73.8%) | 135 (68.5%) |  |
| Slight | 93 (21.6%) | 46 (19.7%) | 47 (23.9%) |  |
| Moderate | 27 (6.3%) | 13 (5.6%) | 14 (7.1%) |  |
| Severe | 3 (0.7%) | 2 (0.9%) | 1 (0.5%) |  |
| Extreme | 0 (0.0%) | 0 (0.0%) | 0 (0.0%) |  |
| Missing | 0 (0.0%) | 0 (0.0%) | 0 (0.0%) |  |
| Anxiety / Depression |  |  |  | 0.395 |
| No | 230 (53.5%) | 124 (53.2%) | 106 (53.8%) |  |
| Slightly | 140 (32.6%) | 80 (34.3%) | 60 (30.5%) |  |
| Moderately | 49 (11.4%) | 26 (11.2%) | 23 (11.7%) |  |
| Severely | 10 (2.3%) | 3 (1.3%) | 7 (3.6%) |  |
| Extremely | 1 (0.2%) | 0 (0.0%) | 1 (0.5%) |  |
| Missing | 0 (0.0%) | 0 (0.0%) | 0 (0.0%) |  |
| Days 3 |  |  |  |  |
| Mobility |  |  |  | 0.131 |
| No problems | 343 (79.8%) | 193 (82.8%) | 150 (76.1%) |  |
| Slight problems | 68 (15.8%) | 31 (13.3%) | 37 (18.8%) |  |
| Moderate problems | 17 (4.0%) | 7 (3.0%) | 10 (5.1%) |  |
| Severe problems | 2 (0.5%) | 2 (0.9%) | 0 (0.0%) |  |
| Unable | 0 (0.0%) | 0 (0.0%) | 0 (0.0%) |  |
| Missing | 0 (0.0%) | 0 (0.0%) | 0 (0.0%) |  |
| Self-Care |  |  |  | 0.571 |
| No problems | 370 (86.0%) | 205 (88.0%) | 165 (83.8%) |  |
| Slight problems | 47 (10.9%) | 21 (9.0%) | 26 (13.2%) |  |
| Moderate problems | 11 (2.6%) | 6 (2.6%) | 5 (2.5%) |  |
| Severe problems | 2 (0.5%) | 1 (0.4%) | 1 (0.5%) |  |
| Unable | 0 (0.0%) | 0 (0.0%) | 0 (0.0%) |  |
| Missing | 0 (0.0%) | 0 (0.0%) | 0 (0.0%) |  |
| Usual Activities |  |  |  | 0.361 |
| No problems | 207 (48.1%) | 117 (50.2%) | 90 (45.7%) |  |
| Slight problems | 132 (30.7%) | 75 (32.2%) | 57 (28.9%) |  |
| Moderate problems | 64 (14.9%) | 30 (12.9%) | 34 (17.3%) |  |
| Severe problems | 21 (4.9%) | 9 (3.9%) | 12 (6.1%) |  |
| Unable | 6 (1.4%) | 2 (0.9%) | 4 (2.0%) |  |
| Missing | 0 (0.0%) | 0 (0.0%) | 0 (0.0%) |  |
| Pain / Discomfort |  |  |  | 0.080 |
| No | 145 (33.7%) | 85 (36.5%) | 60 (30.5%) |  |
| Slight | 183 (42.6%) | 99 (42.5%) | 84 (42.6%) |  |
| Moderate | 82 (19.1%) | 43 (18.5%) | 39 (19.8%) |  |
| Severe | 17 (4.0%) | 4 (1.7%) | 13 (6.6%) |  |
| Extreme | 3 (0.7%) | 2 (0.9%) | 1 (0.5%) |  |
| Missing | 0 (0.0%) | 0 (0.0%) | 0 (0.0%) |  |
| Anxiety / Depression |  |  |  | 0.047 |
| No | 190 (44.2%) | 108 (46.4%) | 82 (41.6%) |  |
| Slightly | 135 (31.4%) | 79 (33.9%) | 56 (28.4%) |  |
| Moderately | 75 (17.4%) | 37 (15.9%) | 38 (19.3%) |  |
| Severely | 26 (6.1%) | 8 (3.4%) | 18 (9.1%) |  |
| Extremely | 4 (0.9%) | 1 (0.4%) | 3 (1.5%) |  |
| Missing | 0 (0.0%) | 0 (0.0%) | 0 (0.0%) |  |
| Week 4 |  |  |  |  |
| Mobility |  |  |  | 0.278 |
| No problems | 270 (81.3%) | 149 (84.7%) | 121 (77.6%) |  |
| Slight problems | 49 (14.8%) | 22 (12.5%) | 27 (17.3%) |  |
| Moderate problems | 12 (3.6%) | 5 (2.8%) | 7 (4.5%) |  |
| Severe problems | 1 (0.3%) | 0 (0.0%) | 1 (0.6%) |  |
| Unable | 0 (0.0%) | 0 (0.0%) | 0 (0.0%) |  |
| Missing | 1 (0.0%) | 1 (0.0%) | 0 (0.0%) |  |
| Self-Care |  |  |  | 0.068 |
| No problems | 308 (93.1%) | 167 (94.9%) | 141 (91.0%) |  |
| Slight problems | 17 (5.1%) | 5 (2.8%) | 12 (7.7%) |  |
| Moderate problems | 5 (1.5%) | 4 (2.3%) | 1 (0.7%) |  |
| Severe problems | 1 (0.3%) | 0 (0.0%) | 1 (0.7%) |  |
| Unable | 0 (0.0%) | 0 (0.0%) | 0 (0.0%) |  |
| Missing | 2 (0.0%) | 1 (0.0%) | 1 (0.0%) |  |
| Usual Activities |  |  |  | 0.152 |
| No problems | 232 (70.1%) | 132 (75.0%) | 100 (64.5%) |  |
| Slight problems | 75 (22.7%) | 35 (19.9%) | 40 (25.8%) |  |
| Moderate problems | 20 (6.0%) | 8 (4.6%) | 12 (7.7%) |  |
| Severe problems | 4 (1.2%) | 1 (0.6%) | 3 (1.9%) |  |
| Unable | 0 (0.0%) | 0 (0.0%) | 0 (0.0%) |  |
| Missing | 2 (0.0%) | 1 (0.0%) | 1 (0.0%) |  |
| Pain / Discomfort |  |  |  | 0.097 |
| No | 170 (51.4%) | 95 (54.0%) | 75 (48.4%) |  |
| Slight | 118 (35.6%) | 66 (37.5%) | 52 (33.5%) |  |
| Moderate | 37 (11.2%) | 13 (7.4%) | 24 (15.5%) |  |
| Severe | 5 (1.5%) | 2 (1.1%) | 3 (1.9%) |  |
| Extreme | 1 (0.3%) | 0 (0.0%) | 1 (0.7%) |  |
| Missing | 2 (0.0%) | 1 (0.0%) | 1 (0.0%) |  |
| Anxiety / Depression |  |  |  | 0.605 |
| No | 167 (50.5%) | 92 (52.3%) | 75 (48.4%) |  |
| Slightly | 98 (29.6%) | 53 (30.1%) | 45 (29.0%) |  |
| Moderately | 51 (15.4%) | 25 (14.2%) | 26 (16.8%) |  |
| Severely | 10 (3.0%) | 5 (2.8%) | 5 (3.2%) |  |
| Extremely | 5 (1.5%) | 1 (0.6%) | 4 (2.6%) |  |
| Missing | 2 (0.0%) | 1 (0.0%) | 1 (0.0%) |  |

^a^ P values of Freeman-Halton tests comparing BNT162b2 cohort and unvaccinated cohort, excluding category of missing.

Supplemental Table 3 Mixed Models for Repeated Measurements EQ-5D-5L and WPAI-GH Scores

|  | EQ VAS | | EQ-5D-5L Utility Index (U.S. weights) | | Absenteeism | | Presenteeism | | Work productivity loss | | Activity impairment | |
| --- | --- | --- | --- | --- | --- | --- | --- | --- | --- | --- | --- | --- |
|  | Coeff (SE) | P value | Coeff (SE) | P value | Coeff (SE) | P value | Coeff (SE) | P value | Coeff (SE) | P value | Coeff (SE) | P value |
| Intercept | 4.51 (5.36) | 0.401 | 0.038 (0.062) | 0.546 | 53.6 (4.9) | <0.001 | 41.4 (5.7) | <0.001 | 57.7 (6.0) | <0.001 | 41.2 (4.9) | <0.001 |
| Vaccinated |  |  |  |  |  |  |  |  |  |  |  |  |
| BNT162B2BNT162b2 | 3.65 (1.46) | 0.013 | 0.069 (0.019) | <0.001 | -19.4 (4.5) | <0.001 | -8.4 (4.2) | 0.047 | -11.2 (4.4) | 0.012 | -6.3 (3.1) | 0.044 |
| No | Reference |  | Reference |  | Reference |  | Reference |  | Reference |  | Reference |  |
| Assessment Time |  |  |  |  |  |  |  |  |  |  |  |  |
| Day 2-4 / Week 1 | Reference |  | Reference |  | Reference |  | Reference |  | Reference |  | Reference |  |
| Week 4 | 9.04 (1.23) | <0.001 | 0.086 (0.014) | <0.001 | -62.9 (3.4) | <0.001 | -30.9 (3.3) | <0.001 | -48.0 (3.6) | <0.001 | -28.9 (2.5) | <0.001 |
| Assessment Time * Vaccinated | | | | | | | | | | | | |
| Day 2-4 / Week 1 * BNT162b2 | Reference |  | Reference |  | Reference |  | Reference |  | Reference |  | Reference |  |
| Week 4 * BNT162B2 | -0.25 (1.69) | 0.884 | -0.025 (0.020) | 0.209 | 22.6 (4.8) | <0.001 | -0.8 (4.4) | 0.863 | 2.8 (4.8) | 0.561 | -4.0 (3.5) | 0.257 |
| Pre-COVID-19 Baseline Score | -0.22 (0.05) | <0.001 | -0.177 (0.057) | 0.002 | -0.8 (0.0) | <0.001 | -0.7 (0.1) | <0.001 | -0.8 (0.1) | <0.001 | -0.7 (0.0) | <0.001 |
| Age, years |  |  |  |  |  |  |  |  |  |  |  |  |
| 18-29 | Reference |  | Reference |  | Reference |  | Reference |  | Reference |  | Reference |  |
| 30-49 | -1.82 (1.45) | 0.211 | -0.011 (0.018) | 0.532 | 3.9 (2.4) | 0.102 | 5.6 (3.0) | 0.065 | 4.1 (3.2) | 0.204 | 7.8 (2.9) | 0.006 |
| 50-64 | -2.84 (1.74) | 0.102 | -0.048 (0.021) | 0.025 | 10.8 (2.9) | <0.001 | 6.8 (3.8) | 0.074 | 7.3 (4.1) | 0.075 | 13.7 (3.5) | <0.001 |
| ≥65 | -1.86 (2.29) | 0.417 | -0.025 (0.028) | 0.367 | 1.7 (5.5) | 0.760 | 0.3 (7.4) | 0.964 | -1.4 (7.9) | 0.863 | 6.9 (4.7) | 0.139 |
| Gender |  |  |  |  |  |  |  |  |  |  |  |  |
| Male | 4.07 (1.30) | 0.002 | 0.028 (0.016) | 0.080 | -2.1 (2.2) | 0.360 | -6.8 (2.9) | 0.019 | -5.8 (3.1) | 0.057 | -9.8 (2.6) | <0.001 |
| Female | Reference |  | Reference |  | Reference |  | Reference |  | Reference |  | Reference |  |
| Race / Ethnicity |  |  |  |  |  |  |  |  |  |  |  |  |
| White (non-Hispanic) | Reference |  | Reference |  | Reference |  | Reference |  | Reference |  | Reference |  |
| Black/African American | 2.17 (2.73) | 0.428 | 0.017 (0.033) | 0.613 | -1.8 (5.1) | 0.726 | 0.8 (6.4) | 0.899 | 0.1 (7.1) | 0.994 | -1.9 (5.6) | 0.730 |
| Hispanic or Latino | 0.65 (1.68) | 0.701 | 0.005 (0.021) | 0.818 | -1.9 (3.0) | 0.530 | 4.3 (3.9) | 0.268 | 4.8 (4.1) | 0.243 | -2.3 (3.4) | 0.494 |
| Asian | 4.24 (2.54) | 0.096 | 0.032 (0.031) | 0.316 | -1.7 (4.7) | 0.713 | 1.4 (5.8) | 0.807 | 2.4 (6.3) | 0.705 | 0.9 (5.2) | 0.858 |
| Patient refused | -0.30 (3.26) | 0.927 | 0.031 (0.040) | 0.438 | -3.3 (6.1) | 0.589 | 1.1 (8.4) | 0.897 | -2.3 (9.0) | 0.800 | -6.4 (7.0) | 0.359 |
| Other | 2.90 (2.81) | 0.303 | 0.019 (0.035) | 0.582 | 3.9 (4.8) | 0.418 | -6.9 (6.5) | 0.288 | -3.1 (6.9) | 0.661 | -7.5 (5.8) | 0.198 |
| Region |  |  |  |  |  |  |  |  |  |  |  |  |
| Northeast | 1.91 (2.00) | 0.341 | 0.015 (0.025) | 0.534 | 6.2 (3.3) | 0.057 | -1.9 (4.2) | 0.648 | -0.5 (4.5) | 0.908 | -2.3 (3.9) | 0.551 |
| South | 0.87 (1.51) | 0.568 | -0.002 (0.019) | 0.905 | -1.0 (2.6) | 0.698 | -2.4 (3.3) | 0.466 | -2.4 (3.5) | 0.507 | -2.9 (3.0) | 0.328 |
| Midwest | Reference |  | Reference |  | Reference |  | Reference |  | Reference |  | Reference |  |
| West | 0.49 (2.08) | 0.815 | 0.019 (0.026) | 0.448 | -0.7 (3.4) | 0.830 | -0.8 (4.5) | 0.857 | -1.3 (4.8) | 0.793 | -3.7 (4.2) | 0.374 |
| Social Vulnerability Index | | | | | | | | | | | | |
| <0.25 | Reference |  | Reference |  | Reference |  | Reference |  | Reference |  | Reference |  |
| ≥0.25 and <0.5 | 1.21 (1.45) | 0.403 | 0.034 (0.018) | 0.060 | 1.3 (2.4) | 0.579 | -3.9 (3.0) | 0.196 | -4.6 (3.3) | 0.158 | -5.2 (2.9) | 0.076 |
| ≥0.5 and <0.75 | 0.93 (1.62) | 0.568 | 0.020 (0.020) | 0.321 | 4.7 (2.8) | 0.090 | -4.8 (3.6) | 0.180 | -6.0 (3.9) | 0.124 | -3.6 (3.3) | 0.276 |
| ≥0.75 | -2.80 (2.14) | 0.192 | 0.009 (0.026) | 0.740 | 2.3 (3.7) | 0.531 | -9.3 (4.7) | 0.048 | -7.8 (5.0) | 0.121 | -5.0 (4.3) | 0.247 |
| Previously tested positive | 0.99 (1.13) | 0.381 | 0.003 (0.014) | 0.816 | -1.9 (2.0) | 0.333 | -0.5 (2.6) | 0.851 | -2.7 (2.8) | 0.329 | -1.2 (2.3) | 0.591 |
| Work in healthcare | -0.90 (1.80) | 0.616 | -0.018 (0.022) | 0.424 | 3.1 (2.8) | 0.272 | 0.8 (3.6) | 0.818 | -0.7 (4.0) | 0.851 | 3.7 (3.6) | 0.300 |
| Work in high-risk setting | -2.83 (1.96) | 0.150 | -0.006 (0.024) | 0.790 | 4.7 (3.4) | 0.166 | 10.3 (4.3) | 0.017 | 13.3 (4.6) | 0.004 | 5.7 (4.0) | 0.155 |
| Live in high-risk setting | 1.36 (2.53) | 0.591 | -0.028 (0.031) | 0.370 | 3.2 (4.7) | 0.502 | 4.8 (6.0) | 0.431 | 7.2 (6.5) | 0.268 | 12.1 (5.0) | 0.017 |
| Immunocompromised | 1.22 (2.61) | 0.639 | -0.018 (0.032) | 0.560 | 13.1 (6.1) | 0.033 | 9.9 (8.4) | 0.243 | 7.9 (9.1) | 0.385 | 5.5 (5.1) | 0.282 |
| Number of Symptom on index day | -0.59 (0.23) | 0.009 | -0.010 (0.003) | 0.001 | 0.8 (0.4) | 0.048 | 1.3 (0.5) | 0.008 | 1.9 (0.5) | 0.001 | 1.8 (0.4) | <0.001 |

Supplemental Table 4 Model Predicting the Missingness at Week 1 and Week 4

|  | Summary: % (n) / Mean (SD) | | | Model | | |
| --- | --- | --- | --- | --- | --- | --- |
|  | Total | Week 1 Missing | Week 4 Missing | Coeff (SE) | P value | Odds Ratio |
| Intercept | 430 | 11.9% (51) | 23.0% (99) | -3.14 (1.19) | 0.009 |  |
| Assessment Time |  |  |  |  |  |  |
| Week 4 | 430 |  | 23.0% (99) | 0.85 (0.12) | <0.001 | 2.3 (1.9, 2.9) |
| Week 1 | 430 | 11.9% (51) |  | Reference | . | 1.0 |
| Vaccination status |  |  |  |  |  |  |
| BNT162b2 | 233 | 12.9% (30) | 24.5% (57) | 0.36 (0.26) | 0.166 | 1.4 (0.9, 2.4) |
| Unvaccinated | 197 | 10.7% (21) | 21.3% (42) | Reference | . | 1.0 |
| EQ VAS on Day 3 | 72.9 (17.5) | 68.9 (19.1) | 71.7 (17.3) | 0.00 (0.01) | 0.967 | 1.0 (1.0, 1.0) |
| EQ VAS change from pre-COVID-19 baseline to Day 3 | -14.4 (14.8) | -18.2 (18.9) | -16.0 (16.6) | -0.01 (0.01) | 0.311 | 1.0 (1.0, 1.0) |
| Age, years |  |  |  |  |  |  |
| 18-29 | 87 | 8.0% (7) | 16.1% (14) | Reference | . | 1.0 |
| 30-49 | 213 | 10.3% (22) | 23.9% (51) | 0.69 (0.36) | 0.052 | 2.0 (1.0, 4.0) |
| 50-64 | 94 | 16.0% (15) | 27.7% (26) | 0.94 (0.40) | 0.019 | 2.6 (1.2, 5.6) |
| ≥65 | 36 | 19.4% (7) | 22.2% (8) | 0.72 (0.52) | 0.161 | 2.1 (0.8, 5.6) |
| Gender |  |  |  |  |  |  |
| Male | 103 | 4.9% (5) | 16.5% (17) | -0.80 (0.32) | 0.013 | 0.4 (0.2, 0.8) |
| Female | 327 | 14.1% (46) | 25.1% (82) | Reference | . | 1.0 |
| Race / Ethnicity |  |  |  |  |  |  |
| White (non-Hispanic) | 295 | 9.8% (29) | 19.7% (58) | Reference | . | 1.0 |
| Black/African American | 20 | 20.0% (4) | 35.0% (7) | 0.67 (0.50) | 0.184 | 2.0 (0.7, 5.3) |
| Hispanic or Latino | 61 | 11.5% (7) | 27.9% (17) | 0.21 (0.36) | 0.565 | 1.2 (0.6, 2.5) |
| Asian | 22 | 18.2% (4) | 27.3% (6) | 0.37 (0.52) | 0.480 | 1.4 (0.5, 4.0) |
| Patient refused | 13 | 30.8% (4) | 30.8% (4) | 0.94 (0.62) | 0.128 | 2.6 (0.8, 8.6) |
| Other | 19 | 15.8% (3) | 36.8% (7) | 1.22 (0.54) | 0.024 | 3.4 (1.2, 9.8) |
| Region |  |  |  |  |  |  |
| Northeast | 52 | 7.7% (4) | 21.2% (11) | 0.50 (0.48) | 0.295 | 1.7 (0.6, 4.2) |
| South | 252 | 13.5% (34) | 24.6% (62) | 0.32 (0.37) | 0.378 | 1.4 (0.7, 2.8) |
| Midwest | 77 | 6.5% (5) | 16.9% (13) | Reference | . | 1.0 |
| West | 49 | 16.3% (8) | 26.5% (13) | 0.44 (0.47) | 0.343 | 1.6 (0.6, 3.9) |
| Social Vulnerability Index |  |  |  |  |  |  |
| <0.25 | 98 | 12.2% (12) | 22.4% (22) | Reference | . | 1.0 |
| ≥0.25 and <0.5 | 164 | 9.8% (16) | 19.5% (32) | -0.22 (0.33) | 0.496 | 0.8 (0.4, 1.5) |
| ≥0.5 and <0.75 | 120 | 12.5% (15) | 25.0% (30) | 0.07 (0.35) | 0.850 | 1.1 (0.5, 2.1) |
| ≥0.75 | 48 | 16.7% (8) | 31.3% (15) | 0.38 (0.45) | 0.397 | 1.5 (0.6, 3.5) |
| Previously tested positive | 167 | 15.6% (26) | 26.9% (45) | 0.45 (0.25) | 0.069 | 1.6 (1.0, 2.5) |
| Work in healthcare | 47 | 12.8% (6) | 19.1% (9) | -0.24 (0.42) | 0.567 | 0.8 (0.3, 1.8) |
| Work in high-risk setting | 44 | 13.6% (6) | 22.7% (10) | -0.25 (0.43) | 0.559 | 0.8 (0.3, 1.8) |
| Live in high-risk setting | 22 | 9.1% (2) | 27.3% (6) | 0.38 (0.54) | 0.484 | 1.5 (0.5, 4.3) |
| Immunocompromised | 19 | 10.5% (2) | 15.8% (3) | -0.33 (0.67) | 0.623 | 0.7 (0.2, 2.7) |
| Number of Symptom on index day | 5.3 (2.6) | 5.4 (2.6) | 5.0 (2.5) | -0.07 (0.05) | 0.188 | 0.9 (0.8, 1.0) |
