# Supplementary material for: Impact of COVID-19 and effects of BNT162b2 on patient-reported outcomes: quality of life, symptoms, and work productivity among US adult outpatients
Source: J Patient Rep Outcomes. 2022 Dec 5;6:123. doi: 10.1186/s41687-022-00528-w (PMC9722994; doi:10.1186/s41687-022-00528-w)
Supplement: Supplementary file 2 — Additional file 2 Supplemental Figure 1 Least-Square Estimates and 95% Confidence Intervals of EQ-5D-5L Scores. Supplemental Figure 2 Summary Results of EQ-5D-5L scores across time periods. Supplemental Figure 3 Least-Square Estimates and 95% Confidence Intervals of WPAI-GH Scores. Supplemental Figure 4 Summary Results of WPAI-GH scores across time periods. [file 41687_2022_528_MOESM2_ESM.pptx]

## Slide 1
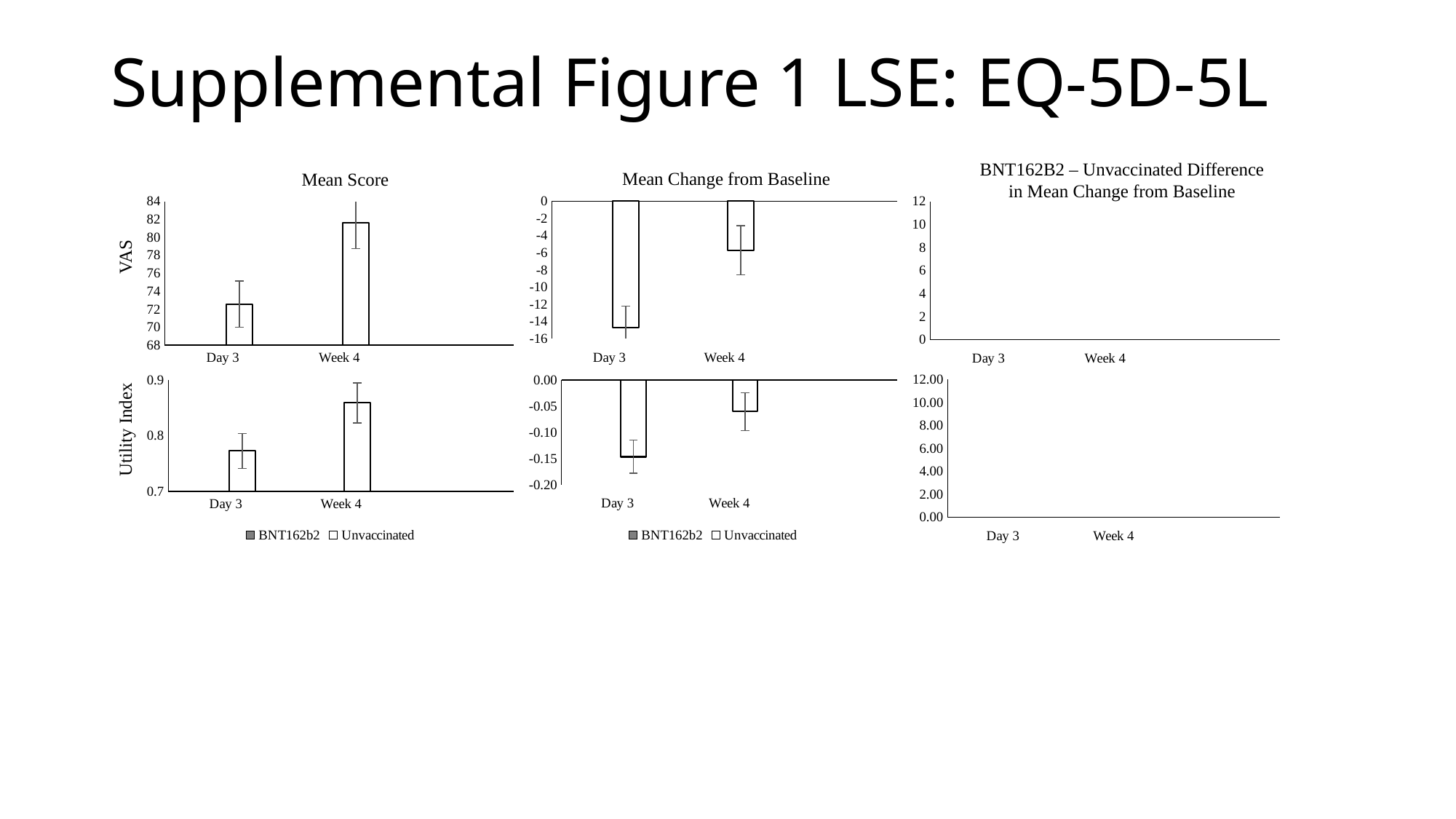

Supplemental Figure 1 LSE: EQ-5D-5L
BNT162B2 – Unvaccinated Difference in Mean Change from Baseline
Mean Change from Baseline
Mean Score
### Chart
| Category | BNT162b2 | Unvaccinated |
|---|---|---|
| Day 3 | 76.2093 | 72.5628 |
| Week 4 | 85.0056 | 81.6057 |
### Chart
| Category | |
|---|---|
| Day 3 | 3.6465 |
| Week 4 | 3.3999 |
### Chart
| Category | BNT162b2 | Unvaccinated |
|---|---|---|
| Day 3 | -11.1138 | -14.7603 |
| Week 4 | -2.3175 | -5.7174 |
### Chart
| Category | Diff |
|---|---|
| Day 3 | 0.06939 |
| Week 4 | 0.04435 |
### Chart
| Category | BNT162b2 | Unvaccinated |
|---|---|---|
| Day 3 | -0.07721 | -0.1466 |
| Week 4 | -0.01596 | -0.06031 |
### Chart
| Category | BNT162b2 | Unvaccinated |
|---|---|---|
| Day 3 | 0.8421 | 0.7727 |
| Week 4 | 0.9033 | 0.859 |VAS
Utility Index

## Slide 2
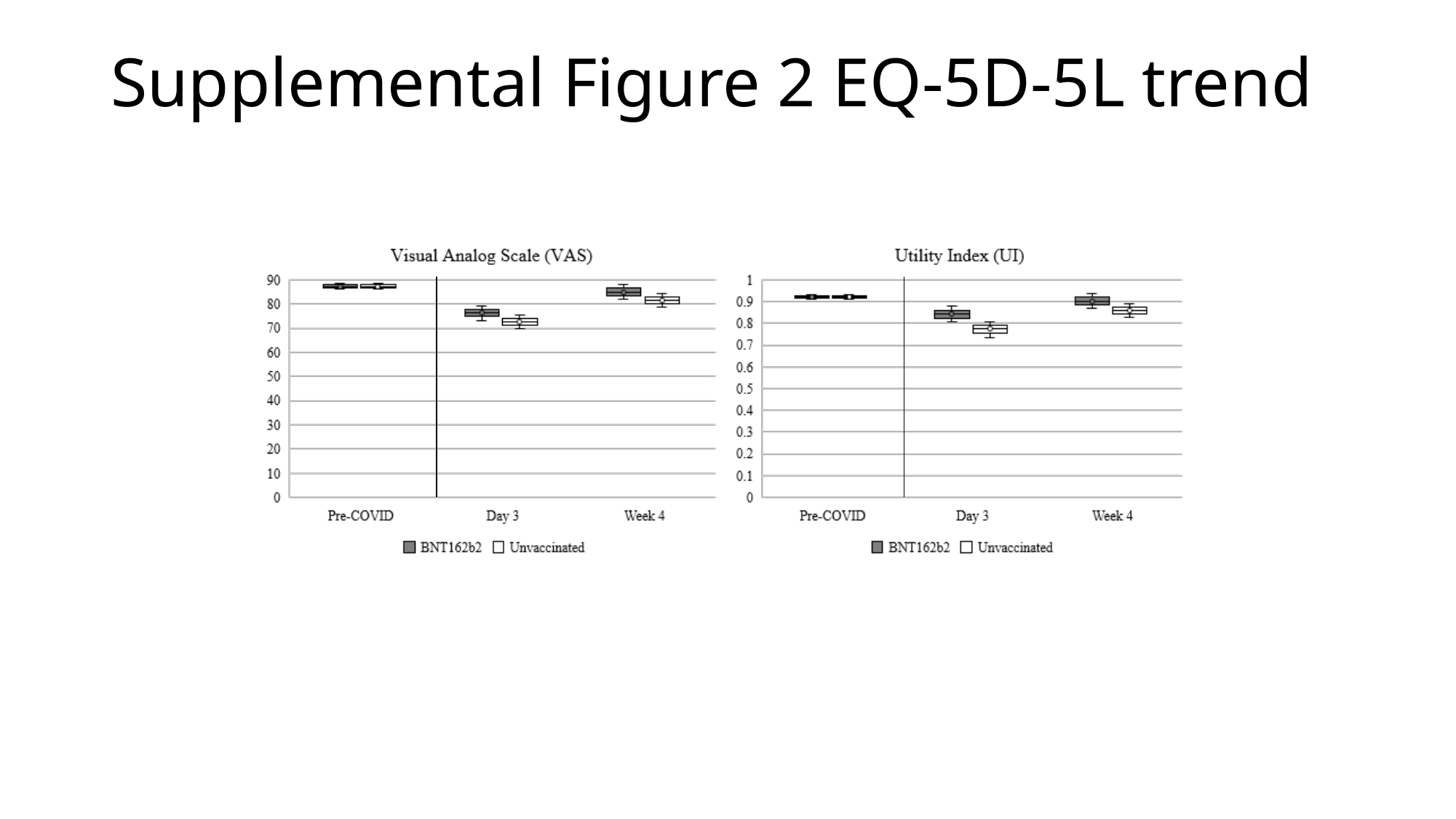

Supplemental Figure 2 EQ-5D-5L trend

## Slide 3
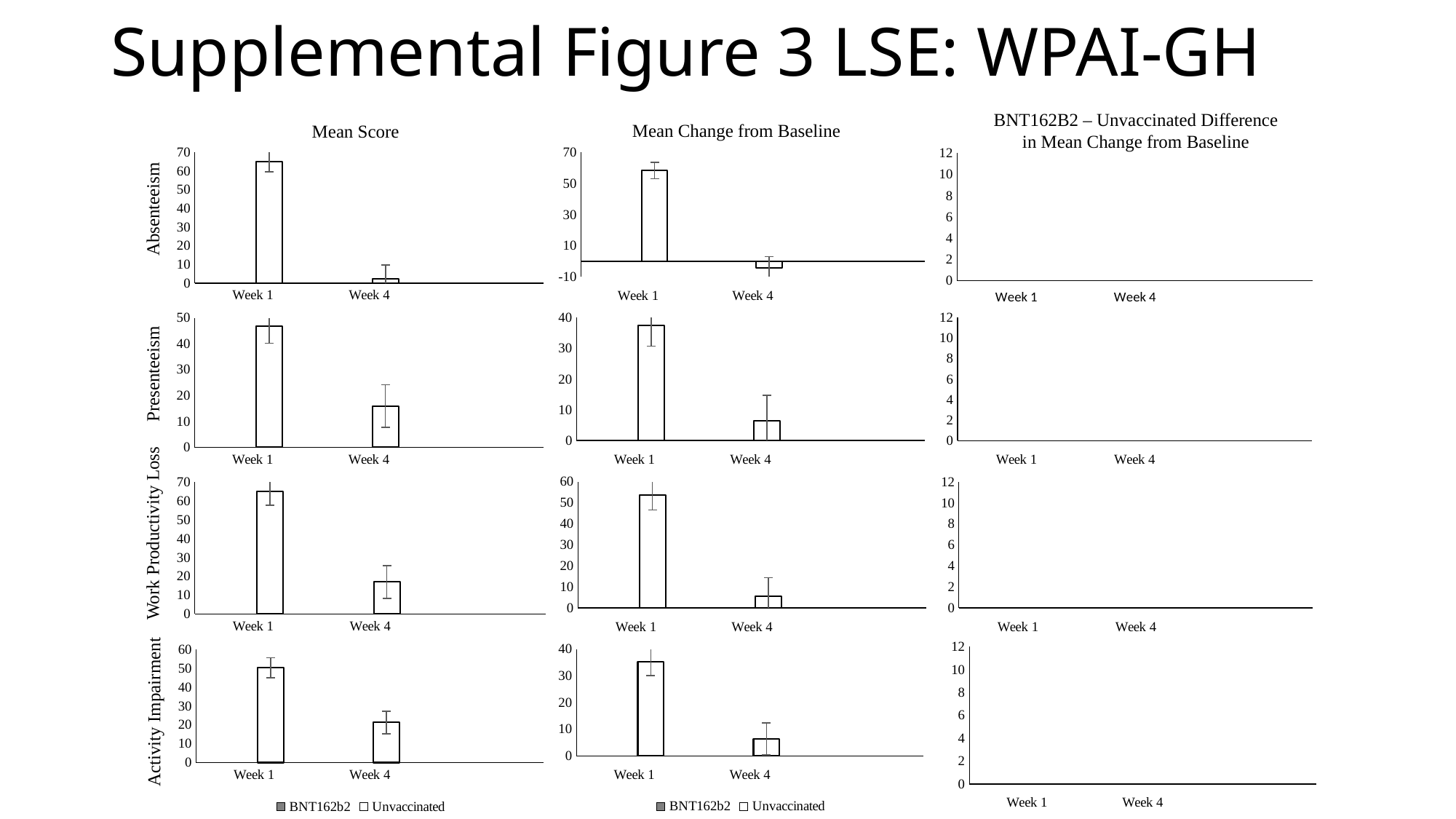

Supplemental Figure 3 LSE: WPAI-GH
BNT162B2 – Unvaccinated Difference in Mean Change from Baseline
Mean Change from Baseline
Mean Score
### Chart
| Category | BNT162b2 | Unvaccinated |
|---|---|---|
| Week 1 | 46.2818 | 64.9827 |
| Week 4 | 5.2817 | 2.126 |
### Chart
| Category | BNT162b2 | Unvaccinated |
|---|---|---|
| Week 1 | 39.7343 | 58.4353 |
| Week 4 | -1.2658 | -4.4214 |
### Chart
| Category | |
|---|---|
| Week 1 | -18.7009 |
| Week 4 | 3.1557 |
### Chart
| Category | BNT162b2 | Unvaccinated |
|---|---|---|
| Week 1 | 28.9659 | 37.3721 |
| Week 4 | -2.6642 | 6.5055 |
### Chart
| Category | |
|---|---|
| Week 1 | -8.4062 |
| Week 4 | -9.1697 |
### Chart
| Category | BNT162b2 | Unvaccinated |
|---|---|---|
| Week 1 | 38.4117 | 46.8179 |
| Week 4 | 6.7816 | 15.9513 |
### Chart
| Category | BNT162b2 | Unvaccinated |
|---|---|---|
| Week 1 | 42.4298 | 53.6313 |
| Week 4 | -2.7763 | 5.6506 |
### Chart
| Category | |
|---|---|
| Week 1 | -11.2015 |
| Week 4 | -8.4269 |
### Chart
| Category | BNT162b2 | Unvaccinated |
|---|---|---|
| Week 1 | 53.7583 | 64.9598 |
| Week 4 | 8.5522 | 16.9791 |
### Chart
| Category | BNT162b2 | Unvaccinated |
|---|---|---|
| Week 1 | 29.0464 | 35.3589 |
| Week 4 | -3.876 | 6.4206 |
### Chart
| Category | |
|---|---|
| Week 1 | -6.3125 |
| Week 4 | -10.2966 |
### Chart
| Category | BNT162b2 | Unvaccinated |
|---|---|---|
| Week 1 | 43.9051 | 50.2177 |
| Week 4 | 10.9828 | 21.2794 |Absenteeism
Presenteeism
Work Productivity Loss
Activity Impairment

## Slide 4
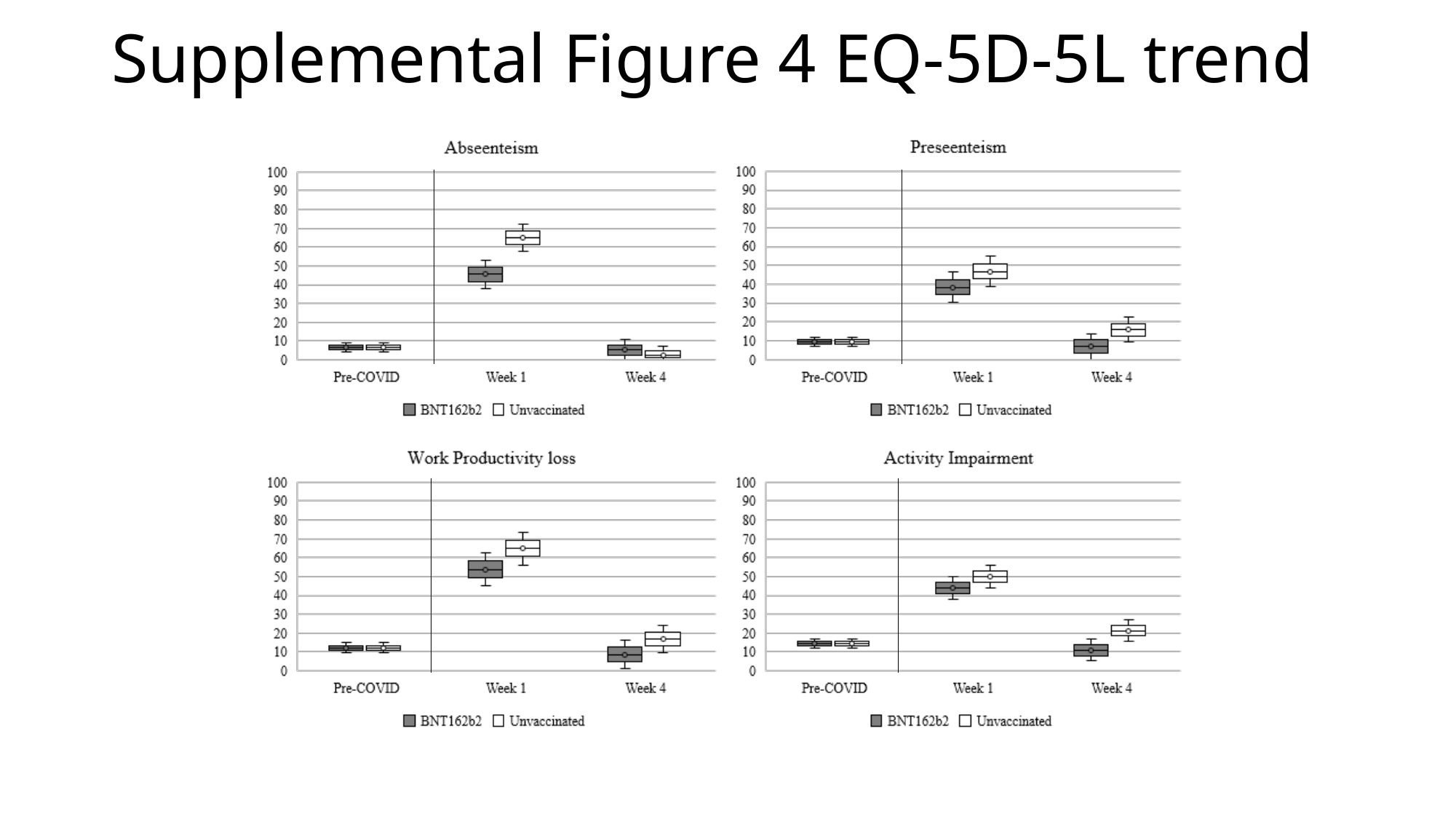

Supplemental Figure 4 EQ-5D-5L trend
